# The Time-Dependent Role of Bisphosphonates on Atherosclerotic Plaque Calcification

**DOI:** 10.3390/jcdd9060168

**Published:** 2022-05-25

**Authors:** Amirala Bakhshian Nik, Hooi Hooi Ng, Manuel Garcia Russo, Francesco Iacoviello, Paul R. Shearing, Sergio Bertazzo, Joshua D. Hutcheson

**Affiliations:** 1 Department of Biomedical Engineering, Florida International University, Miami, FL 33174, USA; abakh002@fiu.edu (A.B.N.); hoong@fiu.edu (H.H.N.); manuel.garcia1405@gmail.com (M.G.R.); 2 Department of Human and Molecular Genetics, Herbert Wertheim College of Medicine, Florida International University, Miami, FL 33199, USA; 3 Department of Chemical Engineering, University College London, London WC1E 7JE, UK; f.iacoviello@ucl.ac.uk (F.I.); p.shearing@ucl.ac.uk (P.R.S.); 4 Department of Medical Physics and Biomedical Engineering, University College London, London WC1E 6BT, UK; s.bertazzo@ucl.ac.uk; 5 Biomolecular Sciences Institute, Florida International University, Miami, FL 33199, USA

**Keywords:** atherosclerotic plaque calcification, calcification paradox, bisphosphonates

## Abstract

Atherosclerotic plaque calcification directly contributes to the leading cause of morbidity and mortality by affecting plaque vulnerability and rupture risk. Small microcalcifications can increase plaque stress and promote rupture, whereas large calcifications can stabilize plaques. Drugs that target bone mineralization may lead to unintended consequences on ectopic plaque calcification and cardiovascular outcomes. Bisphosphonates, common anti-osteoporotic agents, have elicited unexpected cardiovascular events in clinical trials. Here, we investigated the role of bisphosphonate treatment and timing on the disruption or promotion of vascular calcification and bone minerals in a mouse model of atherosclerosis. We started the bisphosphonate treatment either before plaque formation, at early plaque formation times associated with the onset of calcification, or at late stages of plaque development. Our data indicated that long-term bisphosphonate treatment (beginning prior to plaque development) leads to higher levels of plaque calcification, with a narrower mineral size distribution. When given later in plaque development, we measured a wider distribution of mineral size. These morphological alterations might be associated with a higher risk of plaque rupture by creating stress foci. Yet, bone mineral density positively correlated with the duration of the bisphosphonate treatment.

## 1. Introduction

Atherosclerosis represents the most common cause of cardiovascular disease, and atherosclerotic plaque rupture results in arterial thrombosis, leading to heart attack and stroke [1,2]. Vascular smooth muscle cell migration and osteogenic differentiation promote calcification within atherosclerotic plaque [3]. Plaque calcification occurs in 53% and 32% of American male and female atherosclerotic patients, respectively [4]. Comprehensive analyses of coronary artery calcium scores have demonstrated a strong positive correlation between calcification and all-cause mortality, including cardiovascular and coronary artery disease [5]. Although, overall calcium scores positively predict cardiovascular morbidity, local effects of calcification on plaque stability are determined by calcification size and morphology.

Recent clinical studies reported that the percentage calcified plaque volume was a key factor for plaque stability [6]. Plaques with high percentages of calcification were more stable and less likely to rupture. Plaque vulnerability is classically associated with low collagen content in the fibrous cap, which compromises its tensile strength [7]. In silico studies highlighted the presence of destabilizing microcalcifications (5–15 μm)—undetectable due to resolution limits of traditional clinical imaging modalities—in the cap of the vulnerable plaques as a determinant of their biomechanical failure [8,9]. Large macrocalcification (>50 μm) stabilizes the plaque through tissue stress factor reduction. However, microcalcifications destabilize the plaque by creating stress foci within the fibrous cap due to a large mismatch in material properties between the stiff minerals and the surrounding hyperelastic extracellular matrix [8,9,10,11,12]. Prospective clinical data from the Multi-Ethnic Study of Atherosclerosis (MESA) cohort corroborated the biomechanical model predictions linking low density calcification with plaque rupture [12]. These data suggested that coronary artery calcification volume directly contributed to a higher risk of coronary heart disease and cardiovascular events. Interestingly, calcium density inversely correlated with the risk of a cardiovascular event [12]. 

Patients with bone disorders are also at high risk for cardiovascular events [13,14]. Unbalanced bone turnover (either high or low bone formation rate) alters the serum calcium and phosphate levels and increases the risk of vascular calcification [15]. Bisphosphonates (BiPs), the most common anti-osteoporotic strategy, stabilize bone minerals through their high binding affinity to hydroxyapatite and decrease bone resorption by suppressing osteoclast activity [13]. BiPs act as non-hydrolyzable analogs of inorganic pyrophosphate, a common calcification inhibitor in the cardiovascular system [13,16,17]. However, BiPs have demonstrated potential off-target effects on cardiovascular morbidity. BiP usage correlates with a higher risk of cardiovascular/cerebrovascular events in osteoporotic patients with a history of previous cardiovascular events [18,19]. Retrospective clinical trials found paradoxical cardiovascular outcomes in patients taking BiPs. In women with chronic kidney disease, BiPs moderately reduced the hazard ratio of future cardiovascular incidences in patients with no previous cardiovascular events; yet, in patients with prior cardiovascular events, BiPs increased the hazard ratio of future events [20]. 

BiPs may affect vascular calcification through both direct alterations to plaque mineral and systemic effects on calcification mediators. Previous studies reported accumulation of BiPs in the arterial wall of both an atherosclerotic rabbit model and human plaques [21,22]. Short-term BiP treatment elevated serum levels of parathyroid hormone and osteocalcin, key regulators of cardiovascular calcification [23,24], in a female renal failure rat model [25]. BiPs prevented medial calcinosis in a male renal failure rat model; however, this was parallel to a reduction in bone mass density [26]. Apolipoprotein E (ApoE) regulates lipid homeostasis and cholesterol metabolism [27,28], and ApoE deficiency leads to hypercholesterolemia. ApoE-deficient (ApoE^−/−^) mice are commonly used to study mechanisms of atherosclerosis [27]. BiPs altered the mineral microstructure in ApoE^−/−^ mice fed an atherogenic diet, indicating a direct interaction between BiPs and minerals within the plaque [1]. Given the long term administration of BiPs for osteoporosis treatment [29], further studies are needed to assess how BiPs affect vascular calcification over the course of plaque development.

Here, we investigated the effect of BiP treatment on atherosclerotic plaque calcification burden when given at early, intermediate, and late stages of atherosclerosis. We began BiP administration to ApoE^−/−^ mice at three different periods of plaque development based on previous reports [2,30,31] before mineral formation (early), during plaque remodeling and mineral deposition (mid-term), or after the formation of mature, calcified plaques (late stage). Following 25 weeks of an atherogenic diet, we assessed that morphological parameters of total calcification, the average and maximum size of the mineral in treated and untreated groups and correlated the results to bone remodeling. These data might provide insight into the potential cardiovascular-related off-target effects of BiPs.

## 2. Materials and Methods

### 2.1. In Vivo Study

The animal study was approved by the Institutional Animal Care and Use Committee (IACUC) at Florida International University under protocol 17-022 and conformed to current NIH guidelines. Eight-week-old mice with the homozygous genotype for the Apoe^tm1Unc^ mutation (B6.129P2-Apoe^tm1Unc^/J, ApoE^−/−^, *n* = 40, 20 per biological sex) were purchased from the Jackson Laboratory (Bar Harbor, ME, USA). After two weeks of acclimatization and feeding with a regular chow diet, the animals (10 weeks old) were fed with an atherogenic diet (ENVIGO, TD.88137 adjusted calories diet 42% from fat) for 25 weeks. Mice were randomly divided into four groups (5 males and females per treatment): week 5, week 10, and week 15, in which animals received subcutaneous injections of bisphosphonate ibandronate sodium (2 mg/kg/mouse, B1722, APEXBIO, Houston, TX, USA) twice per week (on Mondays and Thursdays), following 5, 10, and 15 weeks of the atherogenic diet, respectively. The control group received a drug vehicle, phosphate-buffered saline (PBS, 1×, PBL01, Caisson Labs, Smithfield, UT, USA). For the injections, animals were partially anesthetized using isoflurane (1% in 2 L.min^−1^ oxygen flow, 07-893-1389, Patterson Veterinary, Loveland, CO, USA). At the study end point (after 25 weeks of diet), the mice received a tail vein injection of a calcium tracer, OsteoSense 680EX (80 nmol/kg/mouse, NEV10020EX, PerkinElmer, Waltham, MA, USA). After 48 h, the animals were anesthetized with isoflurane (1% in 2 L.min^−1^ oxygen flow) followed by retro-orbital bleeding for blood collection. Mice were then immediately euthanized by laceration of the diaphragm before tissue collection. The hearts and aortas were resected and fixed in formalin (10% *w*/*v*, SF100, Fisher Scientific, Hampton, NH, USA) for 2 h. The blood samples were centrifuged at 2000× *g* for 15 min to collect the serum. 

### 2.2. Calcification Morphological Quantification

After resection and removal of excess adventitia and adipose tissues, the aortas were imaged using a near-infrared scanner (LI-COR Odyssey, Lincoln, Nebraska, USA) to visualize the atherosclerotic plaque calcification burden. The signals were localized and quantified using edge detection algorithms in a custom MATLAB (2021b, MathWorks, Natick, MA, USA) script, which quantified the total area of the calcium tracer and normalized to the total scanned aorta area. The resected aortic roots were embedded using Tissue-Plus OCT (23-730-571, Fisher Scientific, Hampton, NH, USA) and cryosectioned (sagittal, 18 μm/section). The serial sections were imaged using a confocal microscopy system (Eclipse Ti, Nikon, Minato City, Tokyo, Japan). To assess total, mean, and maximum calcification area in the aortic root sections (transverse, 18 μm/section), a custom image analysis script was developed in MATLAB. After filtering the background and smoothing the images, individual microcalcifications were identified as connected pixels in binarized images. The script reported the summation of the pixels, average area of the connected pixels (representing the mean of the mineral size), and the maximum connected area (representing the size of the single largest mineral) for each image as total, mean, and maximum calcification area, respectively. Connected areas smaller than 5 pixels were excluded from analyzed data.

### 2.3. Serum Alkaline Phosphatase Activity and Total Cholesterol Assessment

To assess the activity of mouse serum tissue, a non-specific alkaline phosphatase (TNAP), a colorimetric assay kit (K412, BioVision, Milpitas, CA, USA) was used. The samples were diluted 1:20 in assay buffer, and 80 μL of each sample was mixed with 50 μL of 5 mM pNPP solution and incubated for 60 min at 25 °C. The colorimetric change resulting from the reaction was detected using a multi-mode reader (Synergy HTX, BioTek, Winooski, VT, USA) to measure absorbance at 405 nm.

To quantify the serum total cholesterol, a Wako Free Cholesterol E kit (99902601, FUJIFILM Medical Systems USA, Lexington, MA, USA) was used. Briefly, 10 µL of each serum sample was resuspended in 1 mL of assay color reagent and incubated at 37 °C for 5 min. The colorimetric change resulting from the reaction was detected using a plate reader to measure absorbance at 600 nm.

### 2.4. X-ray Computed Tomography (X-ray CT)

Femurs were dissected from mice and imaged directly in a Nikon XT H 225 scanner (macro-CT, Nikon Metrology, Tring, UK). The raw transmission images were reconstructed using commercial image reconstruction software package (CT Pro 3D, Nikon Metrology, Tring, UK), which employs a filtered back-projection algorithm. The scan was performed using 80 kV beam energy, 70 μA beam current, and a power of 5.6 W. A PerkinElmer 1620 flat panel detector was used, with 200 μm pixel size. The resulting effective pixel size was 5 μm. The exposure time per projection was 0.5 s, and a total of 1601 projections were acquired, resulting in a scanning time of approximately 13 min per sample. Bone structural parameters, including thickness and volume fraction (the ratio of bone volume (BV) to total volume (TV)), for both cortical and trabecular regions were assessed using a plug-in module, BoneJ [32], in ImageJ (7.0.13, NIH, Bethesda, MD, USA) [33].

### 2.5. Statistics

Data were presented as the means of independent replications, and error bars represented the standard error of the mean. The reported *n* values represented independent biological replicates. Statistical significance between groups was calculated using two-way ANOVA with Tukey’s post-hoc test model in GraphPad Prism 8. A *p*-value less than 0.05 was considered statistically significant. In the case of comparisons between two groups, the statistical significance was calculated using Student’s *t*-test with *p*-values less than 0.05 considered significant.

## 3. Results

### 3.1. BiP Treatment Increases Atherosclerotic Plaque Calcification in the ApoE^−/−^ Mice Fed an Atherogenic Diet

**Figure 1:**
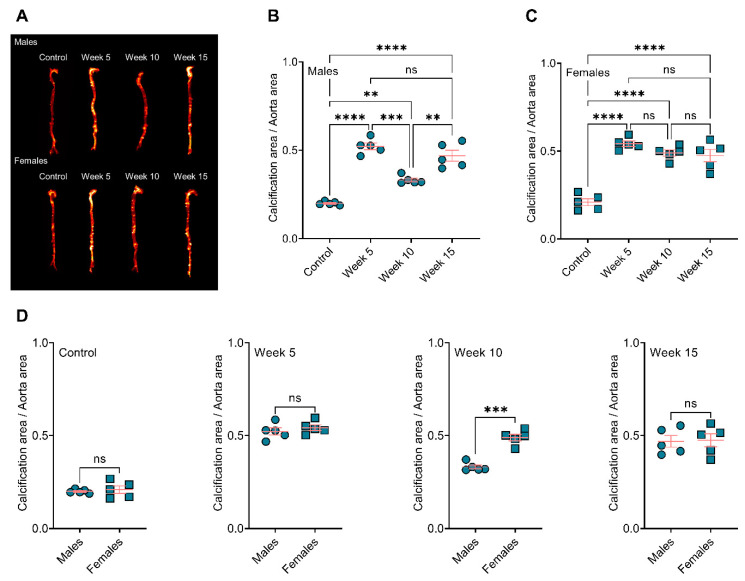
BiP treatment increases the atherosclerotic plaque calcification in ApoE^−/−^ mice fed an atherogenic diet. (**A**) Visualization of the calcium burden using a near-infrared calcium tracer, OsteoSense; (**B**,**C**) quantification of the OsteoSense signal and correlation to total calcification in male and female mice, respectively; (**D**) comparison of total calcification between male and female mice in each group. ** *p* ≤ 0.01, *** *p* ≤ 0.001, and **** *p* ≤ 0.0001, two-way ANOVA with Tukey’s post-hoc test was used for comparison between multiple groups, and Student’s *t*-test was used for comparison between two groups. “ns” stands for not significant.

Visualization and quantification of the calcium tracer OsteoSense from resected aorta revealed significantly elevated plaque calcification in all BiP-treated animals compared to the control group (Figure 1A), regardless of BiP regimen beginning time and sex. In male mice, early (week 5) and late (week 15) BiP treatments showed the highest plaque calcification compared to control and mid-term BiP administration (week 10), as shown in Figure 1B. However, female mice exhibited no significant difference among the BiP treated groups in terms of plaque calcification burden along the aorta (Figure 1C). Interestingly, female mice showed significantly higher levels of calcification compared to males when the BiP treatment began after 10 weeks of the atherogenic diet (Figure 1D). The level of calcification was similar between males and females in the control, week 5, and week 15 groups (Figure 1D). These data demonstrated that BiP may interact with the ectopic atherosclerotic plaque calcification and increase the rate of mineral formation in a sex-dependent manner.

### 3.2. BiP Treatment Alters the Size of Minerals in the Atherosclerotic Plaque

**Figure 2:**
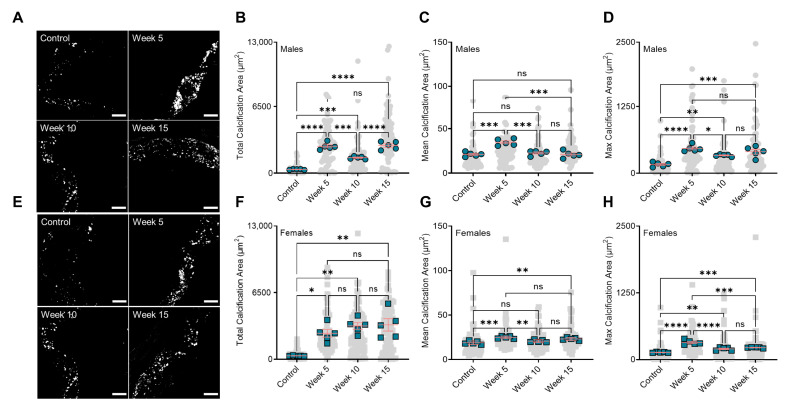
BiP treatment alters the micromorphology of the minerals in the atherosclerotic plaque. (**A**) Visualization of the minerals in the atherosclerotic plaques of male mice (20×, scale bar 100 µm); (**B**) total plaque calcification in male mice; (**C**) mean calcification area (mean of mineral size) in male animals; (**D**) maximum calcification area in male mice; (**E**) visualization of the minerals in the atherosclerotic plaques of female mice (20×, scale bar 100 µm); (**F**) total plaque calcification in female mice; (**G**) mean calcification area (mean of mineral size) in female animals; (**H**) maximum calcification area in female mice. Note that gray points (● or ■) in the background represent technical replications, i.e., all calcifications measured across all mice, and green points (● or ■) represent the biological replications, i.e., the average value for each mouse. * *p* < 0.05, ** *p* ≤ 0.01, *** *p* ≤ 0.001, and **** *p* ≤ 0.0001, two-way ANOVA with Tukey’s post-hoc test. “ns” stands for not significant.

Analysis of mineral morphologies in the aortic roots of the mice showed BiP treatment significantly increased the total calcification area in both male and female groups, compared to the control mice (Figure 2A,B,E,F). Similar to the plaque calcification along the aorta, both week 5 and week 15 male groups had a significantly higher level of total calcification area, compared to the week 10 group (Figure 2B). However, no significant changes were observed between the female BiP-treated groups. The average calcification size was significantly bigger in the early BiP-treated group (week 5) compared to the control, week 10, and week 15 groups. The mean calcification area showed no significant differences between the male control, week 10, and week 15 groups (Figure 2C). In female mice, the mean calcification area increased in both week 5 and week 15 compared to the control and week 10 groups. No significant changes were observed between the control and week 10 groups (Figure 2G). The maximum calcification area and the largest mineral detected in the plaque, increased in all BiP-treated groups compared to the control, regardless of sex. The biggest calcified area in the male groups was detected in both week 5 and week 15 (Figure 2D). In female groups, early BiP treatment increased the maximum mineral size in the plaque compared to other time points, with no significant differences between week 10 and 15 (Figure 2H). Of note is the fact that these data solely reflected aortic root plaque calcification (not aortic valve calcification) due to transverse sectioning of the tissues and the focus of this study, which was atherosclerotic plaque mineralization.

**Figure 3:**
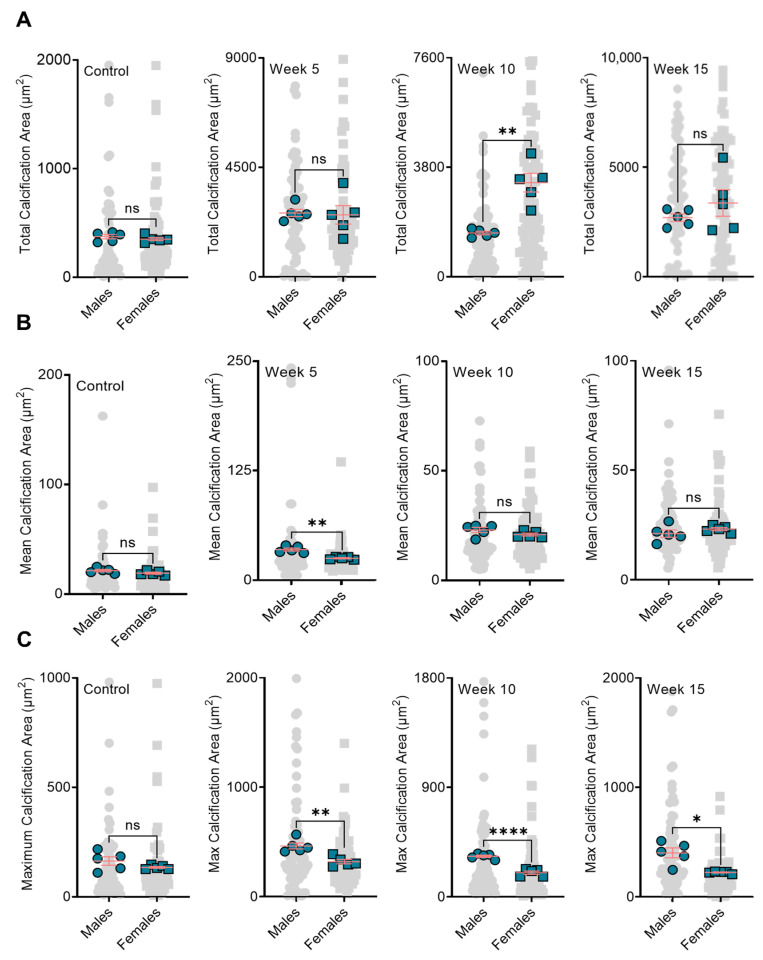
BiP treatment may affect the mineral morphology differently in male and female mouse model. Panel (**A**) is the comparison of total plaque calcification between male and female mice; Panel (**B**) is the comparison of mean calcification area between male and female mice; Panel (**C**) is the comparison of maximum calcification area between male and female mice. Note that gray points (● or ■) in the background represent technical replications, i.e., all calcifications measured across all mice, and green points (● or ■) represent the biological replications, i.e., the average value for each mouse. * *p* < 0.05, ** *p* ≤ 0.01, and **** *p* ≤ 0.0001, Student’s *t*-test. “ns” stands for not significant.

**Figure 4:**
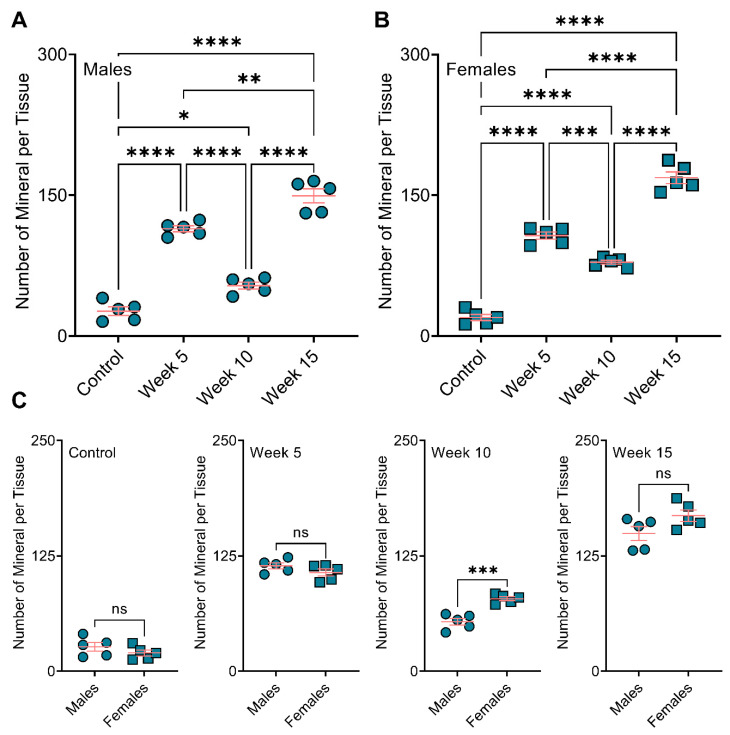
BiP treatment increases the number of minerals in the atherosclerotic plaque of ApoE^−/−^ mice. Number of the mineral per tissue in (**A**) male and (**B**) female mice; (**C**) comparison of total plaque calcification between male and female mice. * *p* < 0.05, ** *p* ≤ 0.01, *** *p* ≤ 0.001 and **** *p* ≤ 0.0001, two-way ANOVA with Tukey’s post-hoc test was used for comparison between multiple groups, and Student’s *t*-test was used for comparison between two groups. “ns” stands for not significant.

When comparing male and female mice, we observed no significant differences in total calcification area in the aortic roots of the control, week 5, and week 15 treatment groups. However, BiP treatment beginning at 10 weeks of diet significantly increased the total calcification area in the female mice compared to the male mice (Figure 3A). The mean calcification area (mineral average size) remained unchanged between the males and females in the control, week 10, and week 15; however, in week 5, the male mice showed a significantly elevated mean calcification area compared to the females (Figure 3B). Regardless of the time at which BiP treatment began, the maximum calcification area in the male groups was higher than that of the females; however, no significant differences were detected in the control group between males and females (Figure 3C). These data suggested that BiP treatment might interact with and alter the morphology of minerals in the plaque. Furthermore, BiP treatment significantly increased the number of individual calcifications within the atherosclerotic plaque both in males and females, compared to control groups. In both male and female mice, week 15 indicated the highest number of calcifications compared to other groups (Figure 4A,B). Comparison between the males and females in each treatment group revealed that in the week 10 BiP treatment group, more mineral was present in the plaque of female mice compare to male ones (Figure 4C).

### 3.3. BiP Treatment Did Not Affect Serum Alkaline Phosphatase Activity and Total Cholesterol

**Figure 5:**
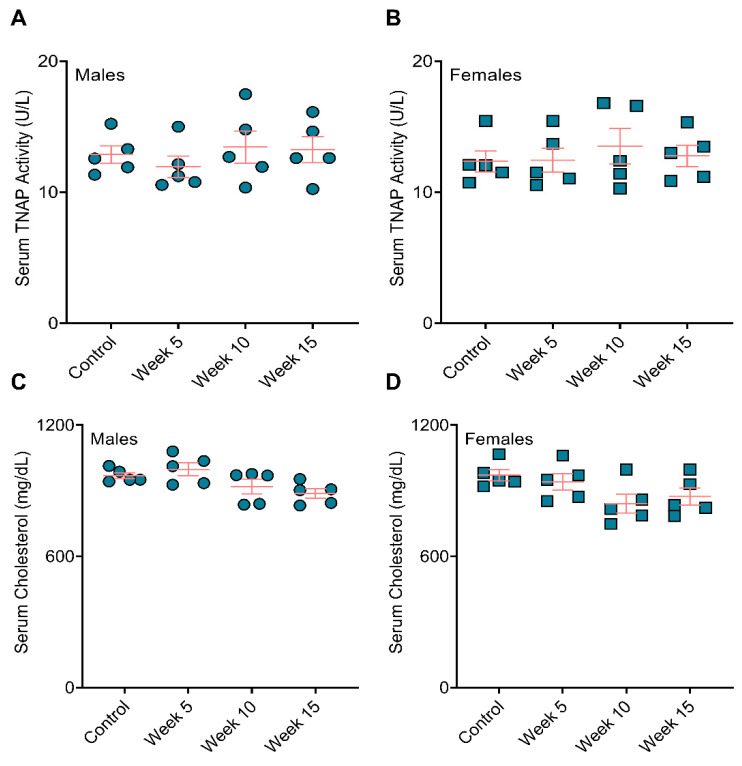
BiP treatment did not affect the serum TNAP activity and total cholesterol in mouse model of atherosclerotic plaque calcification. Serum TNAP activity in (**A**) male mice and (**B**) female mice; serum total cholesterol in (**C**) male mice and (**D**) female mice. No statistically significance observed across the groups, two-way ANOVA with Tukey’s post-hoc test.

We measured the level of alkaline phosphatase, an enzyme required for calcification, in the serum collected at the study endpoint. We observed no significant differences between the BiP-treated and the control groups across either sex (Figure 5A,B). Furthermore, the total serum cholesterol remained unchanged across BiP-treated groups compared to the controls for both male and female mice (Figure 5C,D).

### 3.4. Bone Remodeling Positively Correlated with the BiP Treatment Duration

**Figure 6:**
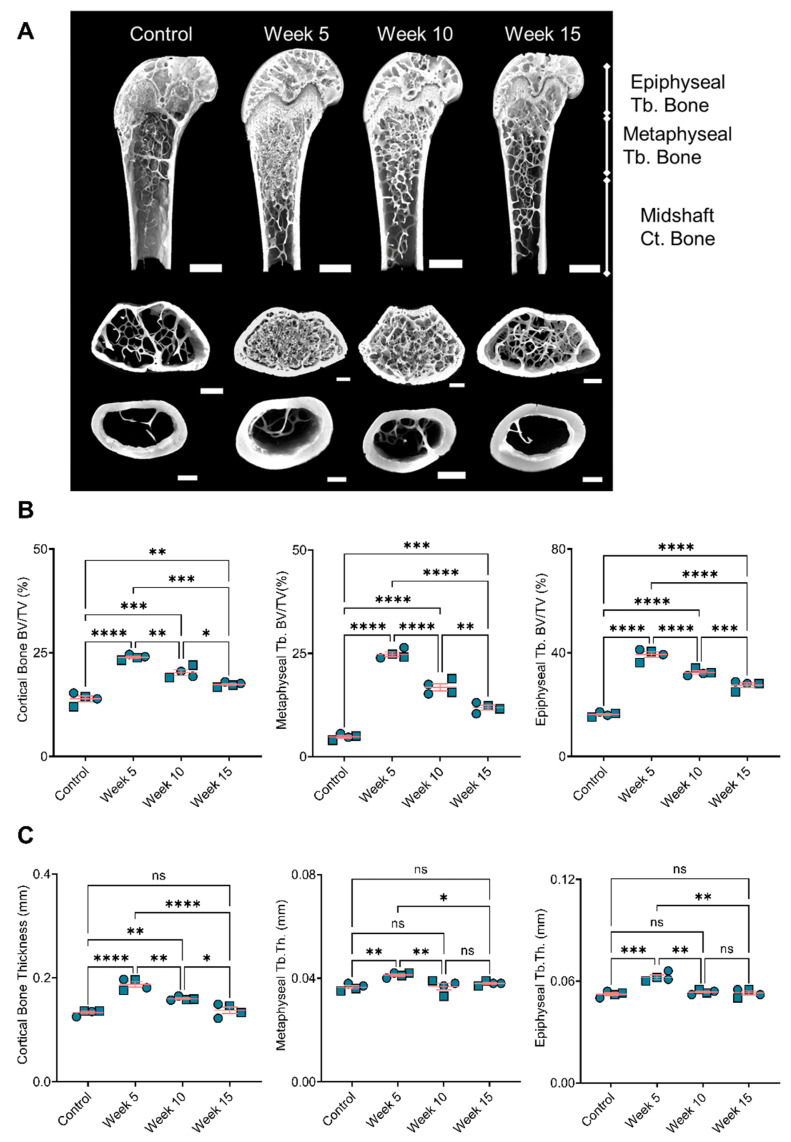
Bone remodeling positively correlated with duration of BiP treatment. (**A**) Reconstruction of femoral bone microstructure for the different treated and untreated groups; Panel (**B**) is the bone volume fraction (bone volume/total volume) for the cortical and trabecular regions; Panel (**C**) is the bone thickness for the cortical and trabecular regions. * *p* < 0.05, ** *p* ≤ 0.01, *** *p* ≤ 0.001, and **** *p* ≤ 0.0001, two-way ANOVA with Tukey’s post-hoc test. Male mice (●) and female mice (■). “ns” stands for not significant.

Analyses of the resected bones showed increased bone volume in BiP-treated mice compared to the control group. The longer treatment with BiP (i.e., beginning the BiP treatment at the early time point) resulted in a higher bone volume in both the cortical and trabecular areas (metaphyseal and epiphyseal regions), as shown in Figure 6A,B. The cortical bone thickness followed a similar trend and exhibited a positive correlation with the duration of BiP treatment. The thickness of the trabecular bone, both metaphyseal and epiphyseal regions, was significantly increased in the longest BiP-treated group (beginning of BiP regimen after 5 weeks of diet); this parameter remained unchanged for the week 10 and week 15 groups compared to the control mice. Table 1 summarizes the bone microstructure parameters analyzed for each group.

## 4. Discussion

Atherosclerotic plaque calcification in ApoE^−/−^ mice fed a chow diet begins around 45–60 weeks of age [34]; however, feeding the mice an atherogenic diet (21% fat and 15% cholesterol) accelerated plaque calcification dramatically following 10–12 weeks of the diet [31,35]. ApoE^−/−^ mice fed an atherogenic diet developed early plaque, including lipoproteins accumulation, immune system activation, and formation of cholesterol-rich foam cells between 4 to 14 weeks of feeding. Lipid cores developed following 14 to 16 weeks of the diet, and fibrous cap formation and mature plaque development occurred over 18 to 20 weeks of the diet [36]. Here, we studied the role of BiP treatments on atherosclerotic plaque calcification in ApoE^−/−^ mice. The animals received BiP treatments twice per week at three different time points: after 5 weeks of the diet, when the plaque formation has started but prior to calcification formation; after 10 weeks of diet, at the onset of plaque calcification; and after 15 weeks of diet, when the plaque was developed and contained calcified regions. 

We showed that the total calcification burden was increased by both the early (starting after 5 weeks of diet and continued for 20 weeks) and late (starting after 15 weeks of diet and continued for 10 weeks) BiP treatment regimens in male mice compared to the control group that did not receive BiP treatment. The early BiP treatment led to a higher mean calcification area (bigger average mineral size) compared to the late treatment, which might stabilize the plaque by reducing the rupture risk. Given the fact that the maximum calcification area was comparable between early and late BiP treatment groups, a smaller mineral size average in the week 15 group meant the presence of a wider range of mineral size distribution, which might influence the plaque stability. Beginning BiP treatment at week 10 resulted in the smallest average and maximum mineral size compared to other BiP-treated male mice, however this was not significantly different from week 15. The female mice showed the same trends regarding average and maximum calcification size, with the smallest average mineral observed in the mice that received BiP treatment beginning at week 10. However, the differences between treatment groups were less pronounced in the female mice. These data suggested that treating mice before calcification began (week 5) resulted in the formation of larger calcifications, whereas starting BiP treatment during early (week 10) or late (week 15) stages of pro-calcific plaque remodeling led to smaller average mineral size. 

Mouse atherosclerotic plaques do not rupture, which limited our ability to perform plaque stability analyses in this study. However, our data suggested that the timing of BiP intervention in relation to ongoing atherosclerotic remodeling could influence mineral morphology and, thus, plaque stability. Previous studies using electron microscopy revealed that timing of the BiP regimen affected the morphology and topography of plaque microcalcifications (less than 5 µm^2^) [1]; starting BiP treatment in early plaque remodeling led to bigger individual mineral aggregates with higher surface roughness, while starting at later time points reduced both mineral aggregate size and surface roughness [1]. Furthermore, mineral aggregates associated with a later BiP treatment start showed qualitatively disorganized and loose morphologies [1]. Finite element-based predictions of these previous data suggested that the microcalcifications associated with all BiP treatment groups would reduce plaque stress compared to non-treated controls. The present data provided a more global analysis of calcification size and burden following BiP treatment and supported the previous observation that BiP altered calcification morphology. 

We showed that starting BiP treatment at early and late stages of calcific remodeling affected the total calcification area similarly in male and female mice. However, for the BiP regimen given during plaque remodeling and mineral deposition onset, the total calcification area was higher in female mice compared to males. The mean calcification area (average of the mineral size) was similar across males and females in this group; yet, male mice had a larger maximum calcification area compared to the females, which suggested a wider mineral size distribution in males. These results might indicate a critical timing in respect of BiP usage. Recent clinical studies indicated a negative correlation between the cumulative BiP dosage (an indication of portion of day covered) to hazard ratio of hospitalization due to atherosclerotic cardiovascular events [37]. Female patients indicated higher hazard ratio (0.958) compared to associated male patients (0.897) [37], an effect that might explain the sex-dependent observation in our week 10 group. Furthermore, these clinical outcomes supported our data that the longer BiP usage duration might lead to larger minerals with a narrow size distribution, which, as in silico studies have suggested, is associated with stable plaque and lower rupture risk [8,38]. 

In humans, 95% of the serum TNAP originates from the bone and liver [39], and unbalanced bone turnover, aging, and chronic kidney disease correlate with abnormal elevated serum TNAP [40,41]. Serum TNAP increases in osteoporotic postmenopausal women compared to postmenopausal women without osteoporosis [40]. BiPs lowered serum TNAP in osteoporotic postmenopausal women and men with heterotopic ossification [40,42,43]. Our data showed unchanged TNAP in serum across the treated and untreated groups. Thus, the observed changes in plaque calcification in all the treated groups appeared to be independent of systemic TNAP changes. Clinical trials reported serum LDL reduction in osteoporotic females treated with BiPs; however, the level of high-density lipoprotein and lactate dehydrogenase remained unchanged [44,45,46]. In the present study, total serum cholesterol remained unchanged in all animals, regardless of the BiP treatment. Our data indicated that the observed calcification differences by BiP treatment were not due to effects on lipid metabolism in ApoE^−/−^ mice.

The outcomes from bone microstructure analyses demonstrated the substantial effect of BiP treatment on bone remodeling. These results revealed a positive correlation between the BiP regimen duration and the bone volume and thickness in cortical and trabecular areas. We showed a positive correlation between bone remodeling and increased plaque calcification for early BiP treatment in ApoE^−/−^ mice. Long term BiP treatment led to increased mineralization in both bone and atherosclerotic plaque. However, for later time points, beginning BiP treatment after 10 and 15 weeks of diet, the data demonstrated a negative correlation between bone remodeling and plaque calcification. Both cortical and trabecular bone volumes were significantly higher in the week 10 group, compared to week 15, while plaque calcification for the latter was significantly higher compared to former. Previous studies reported that BiP might imitate the effects of pyrophosphate, a mineralization inhibitor in the cardiovascular system; however, our data did not support the inhibitory role of BiP to reduce cardiovascular calcification compared to non-treated controls. Importantly, the choice of animal models or patients in clinical trials may affect the BiP outcomes; atherosclerotic plaque calcification represents a complex process in which several cell types, including VSMCs and macrophages [47,48], are involved; thus, the BiP treatment may affect multiple aspects of plaque progression and calcification. Limitations of the current study inclued the small number of mice considered per group and the sole focus on mineral characteristics. Future studies will assess a larger number of mice and investigate changes in cell phenotypes that might influence the formation of calcification within the plaque.

## 5. Conclusions

Atherosclerotic plaque calcification represents a significant predictor of lesion vulnerability. Patients with bone disorders are prone to develop ectopic cardiovascular calcification. Clinical trials correlated BiPs, a common anti-osteoporotic pharmaceutical family, with contradicting cardiovascular outcomes. Here, we demonstrated the importance of treatment timing in BiP-induced mineral disruption or promotion. We indicated that BiP could alter key morphological features of the microcalcifications within the atherosclerotic plaque of ApoE^−/−^ mice, which might determine the risk of plaque rupture. Early beginning of BiP regimen, i.e., before calcification initiates, increased the total calcification in both males and females; however, the treatment led to a narrower mineral size distribution. Later regimen timings, either after mineralization starts or when the plaque is developed, resulted in wider mineral size distribution, which correlated with plaque destabilization and a higher risk of rupture. Interestingly, an early BiP regimen elevated mineralization in both bone and atherosclerotic plaque. However, the BiP regimen correlated negatively with bone mineralization and plaque calcification if given after the onset of plaque mineralization. The outcomes of this study demonstrated that bisphosphonate treatment could alter vascular calcification properties. Further studies are required to determine how these changes in calcification properties affect cardiovascular risk to better inform clinical decision-making.

## Figures and Tables

**Table 1:** Detailed bone structural parameters.

Parameter	Control *n* = 4	Week 5 *n* = 4	Week 10 *n* = 4	Week 15 *n* = 4
**Distal femur**				
Epiphyseal Trabecular Bone				
BV/TV (%)	16.22 ± 0.37	39.3 ± 0.96	32.4 ± 0.5	27.48 ± 0.76
Tb.N (mm^−1^)	7.63 ± 1.11	12.86 ± 0.11	12.82 ± 0.32	12.05 ± 0.34
Tb.Th (mm)	0.052 ± 0.001	0.062 ± 0.001	0.053 ± 0.001	0.053 ± 0.001
Tb.Sp (mm)	0.59 ± 0.06	0.41 ± 0.02	0.42 ± 0.02	0.43 ± 0.02
BS/BV (mm^2^/mm^3^)	0.065 ± 0.002	0.059 ± 0.003	0.057 ± 0.003	0.062 ± 0.003
EF	0.017 ± 0.003	0.033 ± 0.005	0.034 ± 0.009	0.016 ± 0.003
EF_max_	0.885 ± 0.004	0.902 ± 0.005	0.887 ± 0.004	0.88 ± 0.009
EF_min_	−0.82 ± 0.003	−0.825 ± 0.008	−0.812 ± 0.007	−0.81 ± 0.009
DA	1.43 ± 0.05	1.39 ± 0.04	1.42 ± 0.03	1.4 ± 0.07
Metaphyseal Trabecular Bone				
BV/TV (%)	4.82 ± 0.29	24.8 ± 0.5	16.8 ± 0.75	11.86 ± 0.5
Tb.N (mm^−1^)	4.1 ± 0.3	13.1 ± 0.09	11.02 ± 0.07	7.62 ± 0.49
Tb.Th (mm)	0.036 ± 0.001	0.041 ± 0.001	0.037 ± 0.001	0.038 ± 0.001
Tb.Sp (mm)	0.56 ± 0.094	0.29 ± 0.04	0.27 ± 0.015	0.44 ± 0.022
BS/BV (mm^2^/mm^3^)	0.092 ± 0.003	0.085 ± 0.001	0.092 ± 0.003	0.092 ± 0.01
EF	0.12 ± 0.02	0.07 ± 0.005	0.086 ± 0.017	0.07 ± 0.03
EF_max_	0.835 ± 0.019	0.87 ± 0.002	0.86 ± 0.012	0.855 ± 0.03
EF_min_	−0.67 ± 0.036	−0.765 ± 0.013	−0.71 ± 0.022	−0.73 ± 0.05
DA	1.77 ± 0.14	1.6 ± 0.15	1.522 ± 0.09	1.78 ± 0.09
**Femoral midshaft**				
Cortical Bone				
Mean Ct.Th (mm)	0.133 ± 0.002	0.187 ± 0.004	0.159 ± 0.001	0.137 ± 0.005
BV/TV (%)	13.9 ± 0.48	23.9 ± 0.25	20.2 ± 0.6	17.3 ± 0.24
Ps.Pm (mm)	5.65 ± 0.11	5.37 ± 0.2	5.35 ± 0.22	5.33 ± 0.18
Ec.Pm (mm)	4.75 ± 0.11	4.2 ± 0.18	4.15 ± 0.2	4.17 ± 0.21
J (mm^4^)	0.49 ± 0.03	0.455 ± 0.04	0.417 ± 0.007	0.5 ± 0.1

Data were a combination of males and females and collected using X-ray computed tomography of the distal femur and femoral midshaft. Values represent the mean ± SEM. BV/TV = bone volume/total volume; Tb.N = trabecular number; Tb.Th = trabecular thickness; Tb.Sp = trabecular separation; BS/BV = specific bone surface: bone surface area/bone volume; EF = ellipsoidal factor; DA = degree of anisotropy; Ct.Th = cortical thickness; Ps.Pm = periosteal perimeter; Ec.Pm = endocortical perimeter; J = polar moment of inertia.

## Data Availability

All data included in this study will be made available upon request.
